# Correlation of Inflammatory Biomarkers and IgG4 Antibodies with Malaria in Cameroon’s Buea Municipality Children

**DOI:** 10.3390/diseases13040123

**Published:** 2025-04-21

**Authors:** Jerome Nyhalah Dinga, Flora Ayah, Emmanuel Fondungallah Anu, Haowen Qin, Stanley Dobgima Gamua, Anthony Kukwah Tufon, Magloire Essissima Amougou, Rameshbabu Manyam

**Affiliations:** 1 Michael Gahnyam Gbeugvat Foundation, Buea 999108, Cameroon; 2 Biotechnology Unit, University of Buea, Buea 999108, Cameroon; 3 Rollins School of Public Health, Emory University, Atlanta, GA 30322, USA; 4 African Vaccinology Network, Buea 999108, Cameroon; 5 Buea Regional Hospital, Buea 999108, Cameroon; 6 Department of Biochemistry and Molecular Biology, University of Buea, Buea 999108, Cameroon; 7 Department of Microbiology and Parasitology, University of Buea, Buea 999108, Cameroon

**Keywords:** inflammatory biomarkers, chronic inflammation, acute inflammation, malaria, IgG4, α-1-glycoprotein, C-reactive protein

## Abstract

**Background:** In recent decades, malaria has become a major worldwide public health problem in endemic countries, especially with children below five years. Malaria causes inflammation, and inflammatory biomarkers like α-1-glycoprotein (AGP) and C-reactive protein (CRP) are elevated in serum during malaria. This work aimed at assessing the serum levels of AGP (chronic inflammation) and CRP (acute inflammation) biomarkers and IgG4 and their correlation with malaria in children below five years in the Buea Health District of the South West Region of Cameroon. **Methods:** This cross-sectional study was carried out between February and April, 2024. AGP and CRP were measured using Q-7plex Human Micronutrient Measurement Kit while IgG4 levels were measured using Enzyme-Linked Immunosorbent Assay with 80 samples. **Results:** Serum AGP and CRP biomarker levels were significantly higher in malaria-positive children compared to malaria-negative children (*p* < 0.001 and *p* < 0.001, respectively). IgG4 levels were high in malaria-negative children (mean OD = 0.51) compared to children infected with the malaria parasite (mean OD = 0.29), in a manner that was statistically significant (*p* < 0.03). Hemoglobin (Hb) had a strong negative correlation with AGP (−0.62) and CRP (−0.46), meaning that as Hb levels increased, AGP and CRP levels decreased. CRP had a strong positive correlation with both age (0.3) and AGP (0.5), suggesting that as age increased or as AGP levels rose, CRP levels tended to increase as well. **Conclusions:** This study revealed that malaria causes alterations in the serum levels of AGP, CRP, and IgG4 in children below the age of 5 in the Buea municipality of Cameroon. It impacts immune responses by increasing the level of inflammation biomarkers like AGP and CRP and decreasing IgG4, a marker associated with immune regulation. Thus, this study helps the understanding of the inflammatory nature of malaria and could be expanded to aid in the broader public health efforts to control and prevent malaria, reduce its complications, and improve overall health outcomes in children in the Buea municipality.

## 1. Introduction

Malaria continues to be a critical public health challenge worldwide, particularly in Africa and other endemic regions. There were an estimated 268 million cases of malaria globally, with 597,000 deaths attributed to the disease, in 2023 [1]. This persistent burden is proof of the significant public health threat malaria continues to pose, as a leading cause of morbidity and mortality, especially in sub-Saharan Africa.

*Plasmodium falciparum* is by far the most widespread and dangerous species amongst the five known species of *Plasmodium*, especially in Sub-Saharan Africa. In fact, *P. falciparum* is responsible for approximately 95% of all malaria cases in this region, as of 2022 [1]. It is known for its ability to cause severe malaria, which can lead to complications such as cerebral malaria, organ failure, and death, particularly in children and pregnant women [2].

Malaria continues to exact a heavy toll in Cameroon with around 11,000 deaths yearly [3]. In the city of Buea, Cameroon, malaria is endemic with transmission occurring year-round, typically ranging from 1 to 100 malaria cases per thousand individuals annually [4]. Despite various efforts to reduce malaria transmission through interventions such as insecticide-treated nets (ITNs), indoor spraying with insecticides, and antimalarial medications, the disease remains a leading cause of death, especially in rural and underserved communities.

Children under the age of five and pregnant women are particularly vulnerable to malaria [5]. Children in this age group are at the highest risk of developing severe malaria, due to their underdeveloped immune systems. Pregnant women also face increased risks, as malaria during pregnancy can lead to complications such as anemia, miscarriage, stillbirth, and low birth weight [5]. Consequently, ongoing global and regional efforts to combat malaria focus on these high-risk groups through targeted prevention and treatment strategies.

Inflammation in the body is a common response to parasitic infections, serving as a critical defense mechanism in the immune system [6]. When parasites invade host organs, they activate the immune system, triggering a cascade of biological processes designed to fight off the infection. In many cases, the inflammation is a result of the immune system’s attempt to contain and neutralize the parasite, preventing it from spreading further within the body. Malaria, for example, is a parasitic infection that often leads to significant inflammation. The presence of the *Plasmodium* parasite triggers the release of signaling molecules, including damage-associated molecular patterns (DAMPs). These molecules are released from damaged or stressed host cells and act as signals that inform the immune system of the presence of an infection [6] and can cause inflammation.

Inflammation is a defense mechanism by the body; for example, malaria triggers the release of DAMPs which trigger the release of cytokines that promote inflammation and the recruitment of immune cells to eliminate the pathogen [7].

Inflammation during infections has been assessed by measuring biomarkers like α-1-acid glycoprotein (AGP) and C-reactive protein (CRP) [8]. During malaria, tissue damage and immunological activation result in the release of inflammatory cytokines, which in turn boost the liver’s production of AGP and CRP [9], and their levels increase in blood during parasitic infections [10]. CRP levels rise rapidly in response to acute inflammation and decrease quickly once the inflammation subsides while AGP levels rise more slowly than CRP and remain elevated for a longer duration, providing a better indication of ongoing inflammation, including chronic inflammation [10]. Other biomarkers that are involved in parasitic infections include high-sensitivity CRP, procalcitonin, Presepsin, Interleukin-6, N-Outstation Pro-B Type Natriuretic Peptide, soluble urokinase-type plasminogen activator receptor, pancreatic stone protein, CD64, and CD11b [11].

The humoral immune response in malaria is mediated by naturally acquired antibodies against *Plasmodium falciparum* blood-stage surface antigens. These antibodies play an essential role in reducing parasite replication and provide clinical malaria protection. This anti-parasite immunity is gained by repeated exposure to *Plasmodium falciparum* beginning from childhood [12]. Studies have shown that IgG4, a non-cytophilic IgG subtype, does not offer protection against malaria, but during parasitic infection like malaria, IgG4 responses can occur due to the host’s B-cell-mediated and T-cell-mediated immune response. The parasite stimulates specific cytokine production and repression in T-cell induction, leading to a subset of patients where the anti-parasite B-cell response may switch toward IgG4, consisting of over 90% IgG4 [13]. Thus, this research seeks to understand the correlation between malaria and key biomarkers by investigating chronic inflammatory markers such as AGP, acute inflammatory markers like CRP, and serum IgG4 levels, in order to uncover critical insights into the inflammatory response associated with malaria. Understanding these correlations could contribute to more effective diagnosis, treatment, and management strategies for malaria in pediatric populations.

It is hypothesized in this present study that children with malaria in Buea, Cameroon, will show elevated levels of CRP, AGP, and serum IgG4 compared to non-infected children, with a positive correlation between malaria severity and these biomarkers.

## 2. Materials and Methods

### 2.1. Study Design

This is a cross-sectional study that was carried out in the Buea municipality of Cameroon involving the caregivers of children between 0 and 59 months, who were either malaria positive or negative. A malaria-positive child is one whose blood sample contained malaria parasites, as observed under microscopy, and who has a fever (oral temperature > 37.8 °C). In contrast, a malaria-negative child is one who has no fever (oral temperature < 37.8 °C) and no malaria parasites in the blood sample, as confirmed by microscopy.

For the analysis of inflammatory biomarkers, both AGP and CRP were measured. AGP levels were used to evaluate whether chronic inflammation was present, as elevated AGP levels are often associated with prolonged or ongoing inflammation [14]. On the other hand, CRP levels were assessed to determine if an acute inflammation was present with malaria, as CRP is a marker of short-term inflammatory response typically seen in the early stages of an infection [15]. These measurements helped to differentiate between the two types of malaria, providing insights into the duration and intensity of the immune response.

IgG4 is detrimental in malaria with high levels associated with the severity of malaria [16]. This subclass of immunoglobulin G antibody was measured to infer the severity of malaria in the present study.

### 2.2. Study Site and Study Population

Buea has an equatorial climate with temperatures ranging from 25 °C to 29 °C year-round, and it is located at the base of Mount Cameroon. The region experiences two main seasons: the rainy season, which lasts from June to October, and the dry season, which spans from November to May. The study focused on children, both male and female, aged 0–59 months, who were either diagnosed with malaria or served as healthy controls. Malaria diagnosis followed the World Health Organization’s (WHO) 2020 guidelines, which include methods such as microscopy or Rapid Diagnostic Tests (RDTs). For this study, microscopy was used, which involves staining and examining a thick blood smear under a microscope to identify malaria parasites. While Plasmodium falciparum is responsible for 82–100% of malaria cases in Cameroon, depending on the location, no speciation was performed during the microscopy analysis in the present study. The analysis focused solely on detecting the presence of malaria parasites in blood samples.

Children with severe health conditions or those currently undergoing malaria treatment were excluded from the study.

### 2.3. Sample Collection

Blood samples of 4 mL were obtained from each of the 80 participants in this study after parental consent was obtained for each child, through vein puncture. Malaria diagnosis was conducted via microscopy, identifying 43 malaria-positive children and 37 malaria-negative children. Malaria-negative samples were collected from children with no fever and no parasitemia at the point of sample collection. Positive samples were collected from children with fever who had malaria parasites in their blood as determined by microscopy. Each sample was examined by two microscopists. The blood samples were placed in dry tubes and centrifuged at 3000× *g*. The sera were then transferred to Eppendorf tubes and stored at −20 °C for later measurement of IgG4 levels. Additionally, the malaria-positive blood samples were frozen, and whole parasite proteins were extracted from them for use in the ELISA test.

### 2.4. Measurement of AGP and CRP Inflammatory Biomarkers

The 7-Plex Human Micronutrient Measurement Kit was employed to assess the inflammatory biomarkers α-1-glycoprotein and C-reactive protein, adhering to the manufacturer’s guidelines (Quansys Biosciences 2024) [17]. Briefly, to prepare the 1× sample diluent, the 2× sample diluent was mixed with 10 mL of distilled water. One milliliter of the 1× sample diluent was mixed with the lyophilized competitor, left undisturbed for 5 min, thoroughly mixed, and returned to 19 mL of the 1× sample diluent to create the complete sample diluent. Calibrators were reconstituted with the complete sample diluent as specified in the certificate and left to stand for 5 min. The wash buffer was made by diluting 50 mL of 20× concentrate with 950 mL of deionized water, while the chemiluminescent substrate was made by adding 3 mL of substrate A to 3 mL of substrate B. Serum samples were diluted at a ratio of 1:40 with the sample diluent, and 50 µL was added in duplicates onto the pre-coated plates. Controls were included according to the manufacturer’s guidelines. After a 3 h incubation at room temperature, the plates were washed three times with wash buffer, followed by the addition of 50 µL of detection mix and a further 20 min incubation at room temperature. Plates were then washed six more times with wash buffer, and 50 µL of the chemiluminescent substrate was added to each well. Chemiluminescent images of the plate were captured using the BIO-RAD Gel Doc imager (BIO-RAD, Hercules, CA, USA) and analyzed with Q-View software v3.1 (Quansys Biosciences, Logan, UT, USA). The software compared the dot intensities of the analytes in the samples to the calibrated ranges, determining the concentrations of each analyte. Finally, data from two concentrations of each analyte were recorded in an Excel sheet, and their mean value was used for analysis.

### 2.5. Measurement of IgG4 Levels Using the Enzyme-Linked Immunosorbent Assay (ELISA)

ELISA was utilized to identify the levels of IgG4 in the malaria-sick and healthy children as earlier described [18,19] with modifications. ELISA methodology was used because it is sensitive, specific, quantitative, reproducible, reliable, cost-effective, and versatile. Whole malaria parasite lysate in coating buffer was used at 10 µg/mL to coat the ELISA plates overnight. Plates were washed 3 times using 200 µL 1 × TBS-T pH 7.5. A total of 300 µL 2% BSA TBS-T was used to block the plates at RT for 2 h. Naïve sera were used as controls. The plates were incubated with 100 µL of a 1:200 dilution of test and naïve sera in 2% BSA TBS-T for 2 h at RT. The plates were washed 3 times and incubated with 100 µL of a 1:3000 of the anti-human IgG4-HRP in 2% BSA TBS-T for 1 h at RT. After washing 3 times, the antibody–antigen complex was revealed using 100 µL of TMB. Reading of the OD values was conducted using a microplate reader at 450 nm. The absolute OD values were obtained by subtracting the control OD from the test OD values.

### 2.6. Data Analysis

All data were stored in Microsoft Excel and analyzed with SPSS software (version 17.0, Chicago, IL, USA). Statistical techniques such as Student *T*-test (for continuous and numerical data) and Chi-square test (for categorical data) were utilized to assess the statistical significance among covariates between the two groups of the study population (i.e., malaria positive and malaria negative). The R programming language (version 4.2.1) was also utilized. Odds ratios and the association of AGP, CRP biomarkers, and IgG4 levels with malaria were assessed using logistic regression models in Python programming language (version 3.10.11). A *p* value of less than 0.05 was considered statistically significant. Correlation heatmaps, which provide a visual interpretation of pairwise correlations between the explanatory variables in the dataset, were created using the Seaborn library in Python.

### 2.7. Ethical Considerations

The study received ethical approval from the Faculty of Health Science Institutional Review Board (FHSIRB), University of Buea, reference number 2023/2171-10/UB/SG/IRB/FHS of 27 November 2023. The study’s objectives were explained, and the caregiver of each child provided consent by signing an informed consent form.

## 3. Results

### 3.1. Demographic Characteristics of Study Participants

The study analyzed a total of 80 eligible participants, consisting of 43 children diagnosed as malaria positive and characterized by a history of at least 24 h of fever and 37 children who had no fever, were diagnosed negative for malaria, and had no other severe diseases. These malaria-negative children had a mean age of 18.3 months, with a standard deviation of 17.1 months. Demographically, 42 participants (52.5%) were female, while 38 participants (47.5%) were male. In terms of age distribution: 43 participants (53.75%) were aged between 0 and 12 months, 26 participants (32.5%) were in the age range of 13 to 36 months, and 11 participants (13.75%) were aged between 40 and 60 months.

### 3.2. Relationship Between Anemia and Malaria Status

Table 1 shows the relationship between fever, anemia, and malaria status. Hemoglobin levels were categorized with a cutoff value of Hb < 11 g/dL and Hb > 11 g/dL to be anemic and non-anemic, respectively [20]. Malaria was not significantly associated with anemia (*p* < 0.053).

### 3.3. Comparing the Levels of AGP, CRP, and IgG4 in Malaria-Positive and Malaria-Negative Children

The results of the comparison of means of the levels of AGP, CRP, and IgG4 revealed significant differences between malaria-positive and malaria-negative children. In the case of AGP, the mean concentration was significantly lower in malaria-negative children (0.28 g/L) compared to the malaria-positive children (0.55 g/L), with a mean difference of 0.271 (95% CI: −0.411 to −0.132) and a t-value of −3.866 (*p* < 0.001). Similarly, for CRP, the mean concentration was also lower in malaria-negative children (3.02 mg/L) compared to the malaria-positive children (28.61 mg/L), with a mean difference of 25.58 (95% CI: −32.556 to −18.621) and a t-value of −7.312 (*p* < 0.001). However, IgG4 was higher in malaria-negative (mean OD = 0.51) than in malaria-positive children (mean OD = 0.29) in a significant manner (*p* < 0.03). These findings suggest a significant association between malaria and the levels of AGP and CRP biomarkers and IgG4 as seen in Table 2.

**Figure 1:**
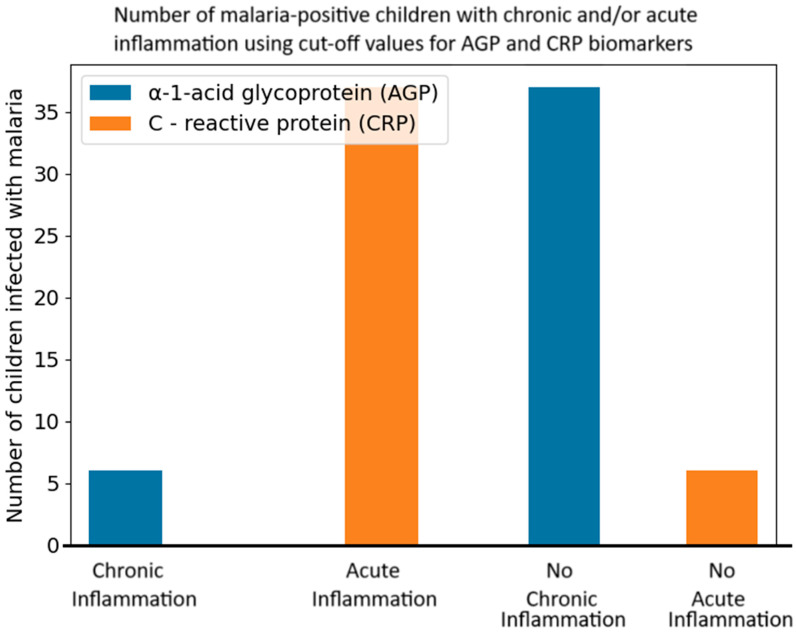
Illustration of inflammation status in children infected with malaria. Children with AGP > 1 g/L were considered as having chronic inflammation [14] while those with CRP > 5 mg/L were considered as having acute inflammation [15].

After categorizing CRP > 5 mg/L to be acute inflammation [21], 37 children were identified to have acute inflammation. In addition, when AGP > 1 g/L was considered as chronic inflammation [22], six malaria-positive children were identified with chronic inflammation. All six malaria-positive children with chronic inflammation equally had acute inflammation, as their CRP values were above 5 mg/L [23]. Only one malaria-negative child had chronic inflammation (AGP = 1.5 g/L) but no acute inflammation (CRP = 2.2 mg/L). The inflammation status of the malaria-positive children is graphically represented in Figure 1.

### 3.4. Assessing the Association Between the Levels of AGP and CRP Biomarkers and IgG4 and Malaria

Multiple regression analysis indicated that malaria significantly increased the chances of having a high serum level of AGP (OR = 46.96, *p* < 0.002). The chances of having a high level of CRP in the serum were slightly increased by malaria. On the other hand, malaria decreased the chances of having high IgG4 levels in the serum of malaria-positive children. However, malaria did not increase the chances of being anemic. There was no effect by sex (OR = 1.123) and age (OR = 1.037) when it comes to being infected by the malaria parasite (Table 3).

### 3.5. Correlation Heatmap of the Study Independent Variables

**Figure 2:**
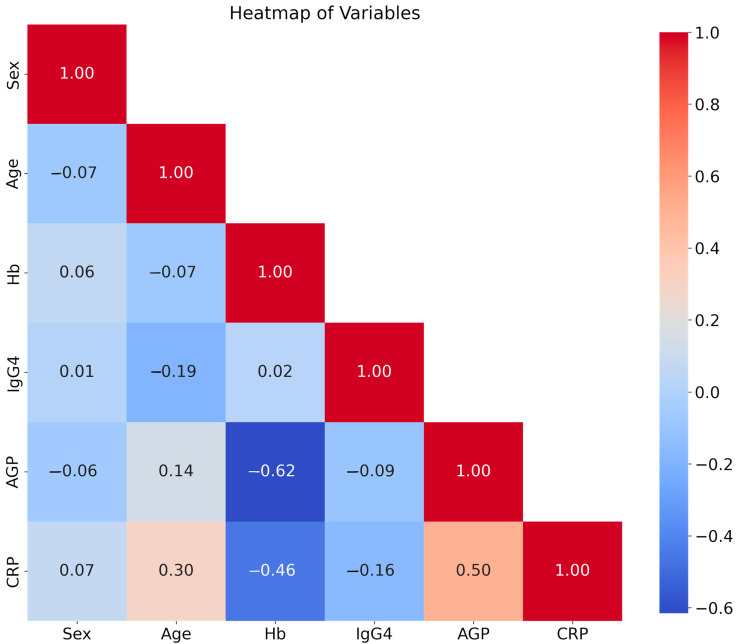
Heatmap of the independent variables IgG4 and inflammatory biomarkers. Python was used to generate a heatmap to show the correlation between the study variables.

Correlation heatmaps demonstrate the correlation between two variables with correlation coefficients ranging from −1 to 1 (where −1 represents a perfect negative correlation, 0 indicates no correlation, and 1 indicates a perfect positive correlation). As shown in Figure 2, each cell in the correlation map contains a number representing the correlation coefficient between two variables. The magnitude and direction of the correlation coefficient imply the strength of the correlation between variables. For instance, hemoglobin and AGP (−0.62) and CRP (−0.46) showed a strong negative correlation, indicating that as hemoglobin levels increased, both AGP and CRP levels tended to decrease. On the other hand, CRP had a strong positive correlation with both age (0.3) and AGP (0.5), suggesting that as age increased or AGP levels rose, CRP levels tended to increase as well. Most other relationships between variables showed weak correlations (values closer to 0), indicating little or no strong association.

## 4. Discussion

Malaria has been shown to induce significant changes in the level of various biomarkers during the course of the infection [24]. One such marker, CRP, has been shown to occur in high levels in children infected with malaria, and this could be due to injury caused by the parasite. This further suggests that some of the children infected with malaria had acute inflammation. Acute inflammation is a typical response to infection, characterized by the activation of immune pathways to combat the parasite and repair damaged tissues. However, after categorizing AGP > 1 g/L as chronic inflammation [25], an interesting observation arose. Six malaria-positive children were found to have chronic inflammation. This further means that the six children had malaria with chronic inflammation. This suggests that they may have been suffering from long-term inflammation. This is consistent with the result of a study by Foote and colleagues [26] who carried out an assessment of inflammatory and immunity proteins during falciparum malaria in children in rural western Kenya.

The six children in question also had acute inflammation indicating a concomitant chronic and acute inflammation. This could present a unique challenge for malaria treatment, which could complicate treatment regimens and hence control of malaria [27]. If malaria caused the chronic inflammation, this would suggest that the disease is not being fully controlled by the immune system, while the acute inflammatory response indicates that there is an ongoing active infection or a recent trauma to the cells of the body. This dual inflammation could complicate the treatment regimens, as it suggests the presence of a persistent or relapsing infection or trauma alongside an immediate inflammatory response [28]. Treating both acute and chronic forms of inflammation simultaneously may require more complex or prolonged interventions. Having both chronic and acute inflammation in these malaria-positive children poses possible long-term health consequences. Complications that can result from chronic inflammation include tissue damage and increase the risk of developing other infections. Moreover, the need for more complex treatment regimens may increase the likelihood of adverse drug reactions or drug resistance [29]. This also highlights the need to monitor and manage inflammation in malaria patients to improve outcomes. This dual inflammatory response highlights the need for a comprehensive approach to malaria treatment that addresses both immediate and long-term health problems [29].

The present study showed a significant odd of having high AGP levels when infected with malaria parasites. This could indicate a heightened inflammatory response, which might help in controlling the infection. However, it also suggests that malaria can significantly impact the body’s inflammatory markers, which could have implications for the diagnosis and management of the disease [30]. Additionally, the presence of chronic inflammation could be an indicator of complications such as tissue damage or organ dysfunction, which could further hinder effective management of the disease. Therefore, understanding and addressing both the acute and chronic inflammation status of malaria are crucial for improving treatment outcomes and controlling the disease in affected populations [31].

Studies have shown that IgG4, a non-cytophilic IgG subtype, does not offer protection against malaria, but during parasitic infection like malaria, IgG4 responses can occur due to the host’s B-cell-mediated and T-cell-mediated immune response [16]. Studies have also suggested that parasitic infection can cause the class switching of immunoglobulin, toward IgG4 production [13]. However, in the present study, IgG4 levels were higher in malaria-negative children compared to the levels in malaria-positive children. This could be because children exposed to malaria may show a distinct immune response profile with elevated levels of IgG4 antibodies compared to individuals exposed to the malaria parasite, potentially due to the absence of malaria stimulation which favors the production of IgG4, while other IgG subclasses like IgG1 and IgG3, which are more crucial for fighting malaria, are produced in lower amounts [16]. This outcome was not in line with results obtained by Mbugi and colleagues [32] who had significant high levels of IgG4 in malaria-sick children compared to healthy children when assessing nutrient deficiencies and potential alteration in plasma levels of naturally acquired malaria-specific antibody responses in Tanzanian children below 5 years. Another possible reason for this difference in results could be the number of samples analyzed. Mbugi et al. [32] analyzed over 300 samples, which was high, compared to the 80 samples analyzed in this study. It is indeed well established that IgG4 levels often rise during malaria, particularly in response to repeated exposure to the parasite. However, IgG4 is not typically considered to play a direct protective role against *Plasmodium*, especially in younger individuals. This is because IgG4 is thought to function differently from other antibody subclasses, such as IgG1 and IgG3, which are more directly involved in immune defense [33] through mechanisms like complement activation and opsonization.

IgG4, rather, is believed to play a role in immune regulation and tolerance. It is often associated with prolonged or repeated infections and is thought to act by dampening excessive inflammatory responses, potentially limiting immune-mediated damage to the host [34]. This can be particularly important in the context of malaria, where repeated infections can lead to a complex and sometimes dysregulated immune response.

Immune biomarkers, such as antibodies (including IgG4), could also reflect the parasite’s ability to persist in the host, especially under suboptimal drug pressures. In the case of prolonged malaria, elevated levels of IgG4, associated with immune tolerance, might allow *Plasmodium* to live longer in the body facilitating the selection of drug-resistant strains. Studies have shown that IgG4 may be linked to prolonged infections and reduced immune activation, factors that could provide a favorable environment for the emergence of drug-resistant parasites [35].

## 5. Conclusions

The present study showed that malaria profoundly impacts immune biomarkers, particularly by increasing the level of inflammation biomarkers like AGP and CRP and decreasing IgG4, a marker associated with immune regulation. These findings underscore the chronic and acute inflammatory nature of malaria and suggest that the immune response to this infection may involve a shift away from immune tolerance or regulation, as seen in the lower IgG4 levels in infected children. These insights into how malaria alters the immune response could have implications for understanding disease progression, immunity, and potential biomarkers for diagnosis and treatment.

Limitations of this study include small sample size, only one *Plasmodium* species being studied, and a comprehensive list of biomarkers not being used, mainly due to resource constraints. Future research should aim to address the sample size limitations of the present study by using a larger sample size for increased statistical power, considering the different *Plasmodium* species, and incorporating a more comprehensive biomarker profile. By expanding on the findings of this study, future work could provide deeper insights into the immune responses to malaria and the role of inflammatory biomarkers, potentially contributing to improved diagnostics, treatment strategies, and vaccine development.

## Figures and Tables

**Table 1:** Relationship between fever, anemia, and malaria status.

		Malaria Status		
Variable	Category	Negative (%)	Positive (%)	*p*-Value
Fever	No	37 (46.3)	0 (0)	**<0.001**
	Yes	0 (0)	43 (53.8)	
Hemoglobin level	Anemic	16 (20)	27 (33.7)	0.053
	Non-Anemic	21 (26.3)	16 (20)	

**Table 2:** Comparison of inflammatory biomarkers (AGP and CRP) and IgG4 between malaria-positive and malaria-negative children.

Biomarker	Malaria	Mean	SD	*p*-Value
AGP (g/L)	Negative	0.28	0.23	**<0.001**
	Positive	0.55	0.37	
CRP (mg/L)	Negative	3.02	7.20	**<0.001**
	Positive	28.61	20.20	
IgG4 (OD value)	Negative	0.51	0.52	**<0.03**
	Positive	0.29	0.36	

**Table 3:** Effect of malaria on the various study parameters.

Variable	Odds Ratio	95% Confidence Interval	*p*-Value	Beta Coefficient
Sex	1.123	[0.465, 2.709]	0.796	0.1160
Age	1.037	[1.005, 1.069]	0.022	0.0362
Hemoglobin	0.766	[0.555, 1.057]	0.104	−0.2672
IgG4	0.304	[0.101, 0.918]	0.035	−1.1898
AGP	46.964	[3.939, 559.958]	0.002	3.8494
CRP	1.207	[1.097, 1.328]	0.0001	0.1883

## Data Availability

The research data are available upon request from the corresponding author.
